# Reasons for Hospitalization of Sickle Cell Disease Patients in the Eastern Province of Saudi Arabia: A Single-Center Study

**DOI:** 10.7759/cureus.19299

**Published:** 2021-11-06

**Authors:** Ossama M Zakaria, Rayan A Buhalim, Faisal A Al Jabr, Mohammed N AlSaeed, Ibrahim A Al-Hajji, Yousif A Al Saleh, Mohammed A Buhalim, Abdulaziz M Al Dehailan

**Affiliations:** 1 Department of Surgery, College of Medicine, King Faisal University, Al-Ahsa, SAU; 2 Department of Medicine, College of Medicine, King Faisal University, Al-Ahsa, SAU

**Keywords:** hip avascular necrosis, hospitalizations, healthcare utilization, hospital readmission, acute chest syndrome, vaso-occlusive crisis, eastern province, saudi arabia, sickle cell anemia, hospital admission

## Abstract

Background: Sickle cell disease (SCD) is among the prevalent chronic diseases in the Eastern Province of Saudi Arabia. To our knowledge, there is no published research that reports the reasons for hospitalization in the Eastern Province of the country. Therefore, this study aimed to fill this gap.

Design and methods: This is a retrospective cohort study that was conducted in the period from January 2018 to December 2019. Patients with sickle cell disease who were admitted and treated in the hospital were included in this study. Patients’ sociodemographic data and reasons for hospitalization were collected and analyzed using the statistical package for social sciences, version 21 (SPSS, Chicago, IL, USA).

Results: There were 103 SCD patients, and the age range was from 18 to 62 years old. The majority of the patients were males (56.3%) and were in the younger age group (≤30 years old; 60.2%). The results showed that the most frequent cause of admission was a vaso-occlusive crisis (VOC) (n=94, 91.3%), followed by acute chest syndrome (ACS) (n=32, 31.1%), and then by hemolytic crisis (27 of the cases; 26.2%). However, we found that a higher number of hip avascular necrosis (AVN) cases were statistically significant in relation to the higher number of hospital admissions (p<0.05), whereas other reasons were not found to have a statistically significant association.

Conclusion: The most frequent cause of admission was VOC episodes, followed by ACS, and then by hemolytic crises. Also, a higher number of hip AVN episodes were statistically significant with the higher number of hospital admissions.

## Introduction

Sickle cell disease (SCD) is an autosomal recessive disorder that is due to a point mutation, which results in a single amino acid substitution in the sixth position of beta chains [1]. It is one of the most common hemoglobinopathies among the Saudi population, especially in the Eastern and Southern regions [2]. There are five haplotypes of the beta globin gene that are associated with sickle cell disease, referred to as Arab-Indian, Benin, Cameroon, Senegal, and Central African Republic. These haplotypes can determine the levels of fetal hemoglobin (HbF) production. With that being said, HbF is an important modulator of the severity of SCD (the higher the levels of HbF, the less severe the disease) [3,4].

SCD is a multisystem disorder that can result in various complications: for example, avascular necrosis, leg ulcers, nephropathy, retinopathy, dactylitis, pulmonary hypertension, high-output heart failure, priapism, gall stones, and hemolytic crises [5]. Moreover, it can result in severe complications that require hospitalization. These complications include acute chest syndrome (ACS), vaso-occlusive crises (VOCs), infections, and strokes [6].

SCD is considered to be a chronic disease, and individuals with frequent admissions to the hospital can incur a lot of medical expenses. As shown in a study conducted among the US population in patients having SCD, medical expenses can reach up to USD $900,000 by age 45 [7]. Apart from the severe complications of SCD that can result in hospitalization, missed appointments, mental health issues, clinic attendance, and financial insecurity are all modifiable risk factors that are associated with frequent admissions [7,8].

Proper identification of the most common reasons for frequent hospitalizations among SCD patients helps in formulating the appropriate guidelines and recommendations by healthcare providers to reduce the frequency of these hospitalizations. There are no previous studies from the literature that have elaborated on the common reasons for hospitalizations in the Eastern region of Saudi Arabia, so the aim of this study was to investigate and study these reasons thoroughly.

## Materials and methods

Data collection and study design

This is a retrospective study conducted in a single secondary hospital located in the Eastern Province of Saudi Arabia. The data were obtained in the period between January 2018 and December 2019 and collected using a printed sheet from the patients' records, including sociodemographic details (age, gender, nationality) and the different causes of hospital admission. Those who were Saudis, admitted in the ward, aged 18 and above, and males and females, who lived in the Eastern region, and who had SCD were included in this study. However, patients who were not admitted and who lived outside the Eastern region were excluded from this study.

Statistical analysis

Quantitative data are presented as mean ± standard deviation (SD) or median with the corresponding interquartile range. Qualitative data are presented as counts and proportions (%). The relationships between the frequency of admission among the baseline characteristics and the different surgical intervention of patients were studied using Fisher’s exact test. A p-value of <0.05 (two sided) was used to indicate statistical significance. Normality tests were conducted using the Shapiro-Wilk test, and a p-value of <0.05 was considered as skewed data. All data analyses were performed using the Statistical Package for Social Sciences, version 21 (SPSS, Chicago, IL, USA).

Ethical considerations

All procedures performed in studies involving human participants were in accordance with the ethical standards of the institutional and/or national research committee and with the 1975 Helsinki Declaration and its later amendments or comparable ethical standards.

## Results

Table 1 presents the baseline characteristics of the recruited patients. A total of 103 SCD patients were included in the study, and the age range was from 18 to 62 years old, of whom 60.2% were in the younger age group (≤30 years). The most common blood type of the patients was type O (53.6%) followed by type B (23.2%) and type A (20.3%). The mean number of times for blood transfusion, vaso-occlusive crisis (VOC), platelet transfusion, and fresh frozen plasma was 3.54, 3.73, 0.72, and 0.22, respectively. Furthermore, the mean level of hemoglobin (Hb), white blood cells (WBCs), platelet, HbS, HbA0, HbA2, and fetal hemoglobin (HbF) was 9.28, 11.3, 333.9, 71.3, 30.2, 3.79, and 19.1, respectively. In addition, the mean length of hospital stay was 19.8 days.

**Table 1:** Baseline characteristics of studied patients. F: fetal. †Not known were excluded from the analysis.

Study variables	Overall
	^(n=103)^
		N (%)
	Qualitative variables		
	Age group	
≤30 years	62 (60.2%)
>30 years	41 (39.8%)
Gender	
Male	58 (56.3%)
Female	45 (43.6%)
Blood group (n=69)^†^	
A	14 (20.3%)
B	16 (23.2%)
O	37 (53.6%)
AB	2 (2.9%)
Quantitative variables	Mean ± SD
Frequency of blood transfusion	3.54 ± 4.12
Frequency of vaso-occlusive crisis	3.73 ± 3.36
Frequency of platelet transfusion	0.72 ± 3.76
Frequency of fresh frozen plasma	0.22 ± 1.51
Hemoglobin level (g/L)	9.28 ± 1.43
White blood cells level (g/L)	11.3 ± 5.07
Platelet level (g/L)	333.9 ± 199.4
Hemoglobin S (%)	71.3 ± 13.8
Hemoglobin A0 (%)	30.2 ± 36.3
Hemoglobin A2 (%)	3.79 ± 4.14
Hemoglobin F (%)	19.1 ± 9.02
Length of hospital stay (days)	19.8 ± 17.5

Table 2 lists the reasons of admission among SCD patients. The most common frequent reason for admission was VOC episodes (n=94, 91.3%), followed by acute chest syndrome (ACS) (n=32, 31.1%) and hemolytic crisis (n=27, 26.2%).

**Table 2:** Reasons for admission.

Reasons parameters	Overall
	^(n=103)^
		N (%)
	Vaso-occlusive crisis		94 (91.3%)
	Acute chest syndrome	32 (31.1%)
Hemolytic crisis	27 (26.2%)
Splenomegaly	16 (15.5%)
Hip avascular necrosis	9 (8.7%)
Hypersplenism	8 (7.8%)
Splenic sequestration crisis	8 (7.8%)
Gall stones	7 (6.8%)
Pneumonia	7 (6.8%)

Table 3 elaborates the reasons of SCD patients’ admission in relation to the frequency of admission. It is found that the higher number of hip avascular necrosis (AVN) episodes was more statistically significant associated with the higher number of hospital admissions (p=0.037), whereas other reasons were not found to have statistically significant association.

**Table 3:** Reasons for admission in relation to the frequency of admission. †P-value has been calculated using Fisher's exact test. *Significant at the p<0.05 level.

Reasons for admission	Overall (n=103) N (%)	Frequency of admission		P-value†
		<5 times (n=67) N (%)	≥5 times (n=36) N (%)	
Vaso-occlusive crisis	94 (91.3%)	59 (88.1%)	35 (97.2%)	0.156
Acute chest syndrome	32 (31.1%)	22 (32.8%)	10 (27.8%)	0.660
Hemolytic crisis	27 (26.2%)	18 (26.9%)	09 (25.0%)	1.000
Splenomegaly	16 (15.5%)	13 (19.4%)	03 (08.3%)	0.165
Hip avascular necrosis	9 (8.7%)	3 (4.5%)	6 (16.7%)	0.037*
Hypersplenism	8 (7.8%)	5 (7.5%)	3 (8.3%)	1.000
Splenic sequestration crisis	8 (7.8%)	5 (7.5%)	3 (8.3%)	1.000
Gall stones	7 (6.8%)	4 (6.0%)	3 (8.3%)	0.693
Pneumonia	7 (6.8%)	4 (6.0%)	3 (8.3%)	0.693

## Discussion

Sickle cell disease (SCD) is a hereditary disorder of an autosomal recessive type. It develops due to a mutation in the beta globin chain caused by a substitution of glutamic acid by valine at the sixth codon [9]. Such a mutation leads to the production of HbS, which is polymerized under hypoxic conditions causing the red blood cells to become sickle shaped, leading to hemolysis, vaso-occlusion, and eventually chronic organ damage [10,11]. The current study discusses the causes of hospitalization of SCD patients in single center. It reports that the most common causes of hospitalization are, in descending order, VOC, ACS, and hemolytic crisis.

Concerning the reasons for admission of SCD patients enrolled in this study (Table 2), it was found that VOC was the most frequent cause of admission, representing a very high percentage (91.3%). This finding is in concordance with a study conducted in Al-Madinah, Saudi Arabia. However, our reported percentage was much higher compared with their study [6]. Although the occurrence of VOC episodes was in equal proportion in both regions, the onset was reported to be earlier in the West than in the East. This complies with delayed development of SCD complications in the East Province [12]. As VOC is the most common reason for admission, patients with SCD should be properly educated about the triggers of the crisis, specifically avoiding excessive physical exertion and extremes of weather and the need for proper hydration [13]. In our study, it is found that the second most common reason for admission is acute chest syndrome, representing 31.1%. This is similar to a previous local study conducted in the West [6]. However, it does not agree with what has been reported previously that the Eastern Province has less frequent acute chest crises [6,12,14]. In the present study, hemolytic crisis was found to be the third cause of hospitalization (26.2%), with a mean hemoglobin (Hb) concentration of 9.28 ± 1.43 g/dL. On the other hand, in a previous Western Saudi study, hemolytic crisis was the fourth cause with a lower mean Hb concentration (8.1 ± 2.1 g/dL) [6]. Splenomegaly poses a risk for splenic complications such as sequestration crisis and hypersplenism. This study suggests that hypersplenism and splenic sequestration crisis were the fourth and sixth leading causes of admission due to SCD, respectively. This corresponds with the fact that splenic complications are more frequent in the Eastern Province than in any other region of the country [12]. Other causes reported were AVN as fifth, gall stones as seventh, and pneumonia as eighth. Interestingly, infection was not reported as one of the causes for hospitalizations. However, other studies in Saudi Arabia by Hawasawi et al. [14] and Abd Elmoneim et al. [6] have reported that infection is considered to be the second and third cause of hospitalization, respectively. Therefore, the findings of this study are consistent with what has been proposed in the theory which states that SCD patients in the Eastern Province have a milder form of the disease, most likely due to the predominance of the Asian haplotype among SCD patients in this area. Conversely, the Benin (African) haplotype, which is in the Western region of the Saudi Arabia, has a severe form [15].

About the reasons for admission compared to the frequency of admission, none of the reasons were statistically significant except AVN of the femoral head that complies with the fact that SCD patients in the Eastern Province (Asian haplotype) have a higher risk of AVN than those in the Western Province (African haplotype) [12,15,16].

Limitations

The current retrospective study might have some limitations. Therefore, a prospective study is needed to avoid any uncertainty of results and closely follow up the patients. Due to the low sample size, the current study findings could not be used to generalize the conclusion across the region. Therefore, further studies with a higher sample size covering risk factors for admission are recommended to enhance the quality of data.

## Conclusions

The current study focused on the experience of a secondary hospital in the area. The study found suggests that the most common reason for admission of SCD patients in the Eastern Province of Saudi Arabia is VOC. However, there is no significant relationship between VOC episodes and the frequency of admission. On the other hand, those who were admitted for more than five times during the studied period have a significant relationship with hip avascular necrosis. The authors recommend a prospective study with the higher number of SCD patients to enhance the quality of data.
